# Complications of Multisystem Inflammatory Syndrome Associated with SARS-CoV-2 Infection—Many Facets of One Disease—A Literature Review Based on a Case Report

**DOI:** 10.3390/jcm13144146

**Published:** 2024-07-16

**Authors:** Aleksandra Stasiak, Piotr Kędziora, Elżbieta Smolewska

**Affiliations:** Department of Pediatric Cardiology and Rheumatology, Medical University of Lodz, Sporna 36/50 Street, 91-738 Lodz, Poland; piotr.kedziora@umed.lodz.pl (P.K.); elzbieta.smolewska@umed.lodz.pl (E.S.)

**Keywords:** MIS-C, SARS-CoV-2, cardiovascular system, child

## Abstract

Multisystem inflammatory syndrome in children (MIS-C) is a disease that made its mark in the early days of the COVID-19 pandemic due to the diverse course and symptoms affecting multiple body systems. It is a condition that develops in pediatric patients about 2–6 weeks after contact with a person infected with the SARS-CoV-2 virus. In many instances, MIS-C has caused multiple organ failure, with particularly severe complications involving the cardiovascular system and manifesting as hypotension, various cardiac arrhythmias, myocarditis or coronary artery lesions resembling those seen in Kawasaki disease. Currently, the incidence of MIS-C is about 1–3 per 1000 children, with a decreasing trend in recent years due to the introduction of immunization against the SARS-CoV-2 virus for children as young as 6 months. In our paper, we present the case of a patient with a severe course of MIS-C with numerous cardiovascular and neurological complications, in whom the symptoms of the disease were managed by administering biological treatment. We also present a review of the literature on the subject, which shows how many different facets this disease can have and that physicians still need to remain alert, as there are cases of severe MIS-C, especially in unvaccinated patients.

## 1. Introduction

Multisystem inflammatory syndrome in children (MIS-C), as defined in 2020 by the World Health Organization, is a condition that affects children and adolescents between the ages of 0 and 19 and is characterized by the presence of fever that persists despite treatment for more than 3 days; the involvement of at least two body systems, of which cardiovascular system involvement appears to be most severe (cardiovascular symptoms include hypotension, shock, features of myocardial dysfunction, pericarditis, valvulitis, or coronary abnormalities); elevated markers of inflammation; the exclusion of an infectious cause of the symptoms; and evidence of current or past SARS-CoV-2 infection (positive PCR test, positive antibody titers, or documented contact with an infected person) [1]. Since the beginning of the COVID-19 pandemic outbreak, definitions of MIS-C have gradually changed due to different variants of the virus, which have altered the clinical presentation of both the infection itself and MIS-C, as well as the introduction of immunization, which is currently available from 6 months of age [2]. A new definition presented by the Council of State and Territorial Epidemiologists (CSTE) and Centers for Disease Control and Prevention (CDC) applicable from 1 January 2023 somewhat simplifies the criteria and adapts them to the current clinical and epidemiological situation. According to the new recommendations, MIS-C criteria apply to patients < 21 years of age, fever of any duration is acceptable, indicators of inflammation currently refer only to CRP values, and two of five organs must be involved (respiratory, renal, and nervous systems were excluded). Symptoms related to the cardiovascular system have now been divided into two categories, the first being shock alone, and the second being other cardiovascular involvement (i.e., reduced myocardial contractility with EF below 55%, coronary artery lesions, or elevated markers of myocardial damage). Criteria from other systems have also been made more specific to particular symptoms, similarly in laboratory tests where the presence of thrombocytopenia and lymphopenia are emphasized. The confirmation of acute or past SARS-CoV-2 infection is still required either by the detection of viral RNA in a polymerase chain reaction (PCR) test or antigen test within 60 days after illness or a positive SARS-CoV-2 serology result (antigen specificity is not required). In the absence of a positive laboratory test result for SARS-CoV-2, the definition of a probable case of MIS-C is met by an epidemiological link, defined as close contact with a probable or confirmed case of COVID-19 within 60 days of illness, and the required clinical criteria [3,4]. Various global organizations have presented their own definitions of MIS-C, which have also changed over the course of the pandemic’s evolution, but in each, the diagnosis is based on the patient’s age, the presence of fever, elevated laboratory markers of inflammation, confirmed organ or system dysfunction, confirmed contact or history of SARS-CoV-2 infection, and the absence of any other cause of the presenting complaints [5]. The main goal of MIS-C treatment is to stabilize the patient’s condition and eliminate life-threatening symptoms, such as shock or heart failure, and prevent distant complications like myocardial fibrosis and scarring or myocardial ischemia due to the formation of a thrombus in a coronary artery aneurysm (these are also the two most common causes of mortality in MIS-C) [2]. Most studies regarding MIS-C emphasize the impact of the disease on the cardiovascular system. Cardiovascular changes include left ventricular dysfunction, dilatation or aneurysms of the coronary arteries, conduction disturbances, or, less commonly, valvular dysfunction and pericarditis and can occur in up to 80% of MIS-C patients [2,3].

## 2. Literature Review

### 2.1. Epidemiology

The incidence of MIS-C, according to various sources, is currently 1 per 1000 children, compared to about 3 per 1000 at the beginning of the COVID-19 pandemic; it is a condition attributed to the introduction of immunization and mutations of the virus [6]. Cardiovascular involvement in patients is most often responsible for the severity of the MIS-C course. Patients diagnosed at the beginning of the pandemic were more likely to present symptoms of circulatory failure and require hospitalization in intensive care units. In subsequent COVID-19 waves, the phenotype of Kawasaki’s disease (KD) was more frequently observed, but with rare involvement of the coronary artery lesions. Nevertheless, one-quarter of the patients still presented symptoms of shock, and one-third required intensive treatment in the PICU [7].

### 2.2. Pathogenesis

As for the origin of the disease itself and the contributing factors that seem to be decisive of the severity of the course, an immunological substrate is considered, or more specifically, the overexpression of the immune response, especially in genetically susceptible individuals. The literature emphasizes the role of elevated titers of interferon-gamma; interleukin (IL)-1, 8, 10, and 17; and tumor necrosis factor-a [8]. Zhao et al. decided to analyze inflammatory markers in MIS-C and compare them in patients with severe and less severe COVID-19 and MIS-C. The authors demonstrated that younger children showed significantly decreased inflammation in laboratory tests than older children. The authors report that these differences may be due to exposure to SARS-CoV-2 infection and the differences in the nasal expression of angiotensin-converting enzyme 2, which is the receptor of SARS-CoV-2 infection, in different age groups. This receptor is also present in the myocardium, which supports the predisposition to myocardial involvement and damage to myocardial contractility, as well as an increase in markers of myocardial damage [5,9]. A paper evaluating lymphocyte subsets in seven children with MIS-C revealed marked decreases in CD4+ and CD8+ T cells with a higher CD4+/CD8+ T-cell ratio. The authors report that T-cell levels normalize after SARS-CoV-2 infection after 2 weeks, and prolonged post-infection lymphopenia may be a marker of inflammatory response and the development of MIS-C [10]. The hyperinflammatory state observed in MIS-C is caused by a dysregulated innate immune response of the host. Long persistent viremia in children (SARS virus can be found in stools even weeks after infection) in predisposed individuals (HLA, genetic background) can trigger an excessive immune response. Macrophage activation and endothelial damage is underpinned by cytokine storm and B-cell tolerance [11]. A whole-exome sequencing study conducted on Brazilian children in 2022 revealed 10 very rare mutations in eight genes, which seem to predispose the body to a less effective immune response to SARS infection or a delayed hyperimmune response [12]. The mechanisms and pathophysiology of their development are still not fully understood. Damage to cardiomyocytes by SARS-CoV-2 virus and vascular wall damage caused by an abnormal immune response are reported [2,13].

### 2.3. Treatment 

The treatment of MIS-C is mainly based on immunomodulatory therapy in the form of intravenous infusions of human immunoglobulins in a dose of 2 g/kg and intravenous corticosteroids in a dose of 1–2 mg/kg/day. Currently, the administration of a second dose of IVIG is not recommended due to the risk of hemolytic anemia and cardiovascular volume overload (especially in patients with impaired left ventricular function). The literature and treatment guidelines for MIS-C issued by the American College of Rheumatology (ACR) and updated in the year 2022 also highlight the role of the interleukin-1 blocker (anakinra) as an important drug for severe MIS-C with circulatory involvement and emphasize the role of IL-1 as the main agent responsible for the cytokine storm and severe inflammatory reaction [2,14,15]. Unfortunately, at present, there are no specific indications, doses, or durations of use for biologic agents; however, anakinra in a high dose of >4 mg/kg/day administered intravenously or subcutaneously should be considered as immunomodulatory therapy in patients who are refractory to previous treatment or in those who have contraindications to glucocorticosteroids. Some patients with a more severe course may require immunomodulatory therapy for 2–3 weeks or even longer [13,14]. The effectiveness of anakinra seems to be confirmed by its inclusion as a subsequent stage of MIS-C treatment in the guidelines and by several case reports.

Extracorporeal membrane oxygenation (ECMO) is a method of advanced life support in patients with severe cardiovascular and respiratory diseases unresponsive to optimal conventional management. Indications for ECMO include congenital structural heart defects or acquired heart disease, such as myocarditis, as well as impaired gas exchange and respiratory acidosis. The selection of patients eligible for ECMO should be made with caution to avoid complications such as liver failure, renal failure, intracranial hemorrhage, or cardiac tamponade [16]. Miller, in his study of the use of ECMO support in hemodynamically compromised patients, found similar results of this treatment and its safety in patients with and without MIS-C [17]. Schwartz et al. described the case of a 5-year-old child with severe left ventricular dysfunction who qualified for ECMO therapy, with good results of this treatment [18]. 

### 2.4. Changes in Cardiovascular System—Diagnosis, Follow-Up, and Current Knowledge

ACR guidelines report myocardial involvement with myocardial dysfunction in up to 55% of patients and coronary lesions in about 20% of patients with MIS-C. After 30 days from the onset of symptoms, these lesions regress in about 80% of patients. Arrhythmias and conduction abnormalities are less common than structural changes in the heart but can include conduction blocks, ventricular and supraventricular arrythmias, rapid heart rhythms, or Brugada-like recordings [15,19,20]. Resting ECG recordings also show abnormal repolarization and ST-T segments, as well as the prolongation of the QT interval [21]. Younger children are more likely to present features of KD with/or without lesions in coronary arteries, and adolescents are more likely to develop shock and features of myocarditis and ventricular dysfunction; however, children in each age category may develop coronary artery aneurysms [15]. In their review on cardiac involvement in MIS-C published in 2021, Alsaied et al. state that even up to 95–100% of patients have elevated troponin or NT-proBNP [21]. Recommendations for the diagnosis and control of cardiovascular symptoms in the ACR guidelines show a high level of consensus for the control of laboratory parameters related to cardiac function and damage (troponin, BNP) until they normalize; echocardiographic studies performed at diagnosis and in follow-up should include the assessment of ventricular/valvular function, pericardial effusion, and coronary artery dimensions with measurements indexed to body surface area using z-scores; additional imaging studies in patients with significantly impaired left ventricular function (EF < 50%) should include cardiac MRI performed in patients 2–6 months after MIS-C diagnosis. Moderate to high consensus was reached on the statements that electrocardiographic recordings should be performed every 48 h in case of conduction disturbances, such patients should be monitored during hospitalization, and Holter ECG recordings are recommended in outpatient care. Echocardiograms should be repeated 7–14 days after diagnosis, and then 4–6 weeks after the onset. Patients with persistent cardiac abnormalities, whether in the coronary arteries or in the form of left ventricular dysfunction, should remain under ongoing cardiac care depending on their condition, and patients in whom the lesions have withdrawn should have an echocardiogram one year after MIS-C [15]. Although myocardial lesions resolve relatively quickly, the distant complications of MIS-C are still unknown and may include myocardial fibrosis, so the ongoing follow-up of these patients and periodic cardiac MRI is important [15].

Most information on the course of MIS-C has been obtained from case reports and single-center studies, but the follow-up time is still too short to predict distant complications. A single-center publication from Bulgaria reports that among 51 patients with MIS-C, 53% had cardiovascular involvement, including pericardial effusion in 33% and pericarditis without effusion in 38%, as well as myocardial dysfunction arterial hypotension, heart failure, myocarditis, coronaritis, and ECG abnormalities. Pericarditis was observed more frequently in patients with elevated CRP and IL-6. This study confirmed that even asymptomatic patients can develop cardiovascular involvement, so every patient with MIS-C should have cardiac screening [22]. Another single-center study from Poland, which presents 51 patients with MIS-C hospitalized over a course of one year, reports that as much as 31% of the study group required transfer to the ICU due to severe hypotension or myocardial hypokinesis visualized in more than one-quarter of the patients. Pericardial fluid was visible in echocardiographic examination in nearly 83% of patients. Mild to moderate valvular dysfunction was found in 96% of patients. Changes in the coronary arteries in the form of the widening of these vessels or hyperechoic arterial walls affected as many as 80% of patients. At follow-up 6 weeks after the onset of the disease, only trace regurgitation of the heart valves was observed in 49% of patients, pericardial fluid was observed in 47%, and coronary artery lesions were found in 19.6%. Completely normal echocardiographic and electrocardiographic images were found in 25.5% of patients. This proves that promptly implemented proper treatment yields good clinical results, and patients should remain under periodic cardiac follow-up [23]. A nationwide surveillance study from Switzerland based on 204 patients also confirms that most of the cardiovascular changes resolve within a few weeks from the onset of the disease; however, cardiovascular involvement is the main concern and focus in MIS-C, especially in patients with involvement of coronary arteries [24]. Ludwikowska et al. collected a registry of nearly 500 MIS-C patients from 50 cities in Poland, in which up to 91% of patients had cardiovascular changes. Atrioventricular valve regurgitation was the most common finding, followed by reduced myocardial contractility, which improved within a few days of treatment initiation (average increase in EF of 10% within 5 days), and coronary artery lesions were less common [25]. Ludwikowska et al. also showed that the characteristics of the course of MIS-C can be distinct to a region in which it occurs, and treatment should be individualized. The incidence of MIS-C depends on gender (boys), age (pubertal age), and ethnicity. Symptoms suggestive of KD (rash, conjunctivitis, lymphadenopathy) were the most common symptoms observed, followed by gastrointestinal lesions. Decreased ejection fraction was observed in 22.7% of children, aneurysms in 8.2%, and pericardial effusion in 9.4%. Older boys were more likely to have myocardial symptoms. Neurological symptoms included lethargy (59.4%), irritability (41.7%), headache (46.1%), and photophobia (11.0%). Peripheral muscle tone abnormalities were not reported. In 8.4% of patients, hospitalization in the PICU was required; no patient required ECMO [26].

In 2020, Dufort et al. described a group of 191 patients with suspected MIS-C from 106 New York State hospitals. In this study group, the male sex predominated, children aged 6–12 years were most often affected by the disease, and in addition to fever, the most common symptoms involved the gastrointestinal tract and dermatological lesions. Depending on the age group, there was a positive PCR for SARS-CoV-2 infection in 46–62% of patients, while there were positive IgG class antibodies in almost 100%. In this report, as many as 80% of patients required hospitalization in the ICU, and 10% required mechanical ventilation. In addition to standard treatment (IVIG and glucocorticosteroids), as many as 62% required vasoconstrictive drugs. As for cardiovascular lesions, 52% of patients developed left ventricular dysfunction, 32% pericardial fluid, and 9% coronary artery lesions. Kawasaki disease was diagnosed in 36% of patients, and also, 36% of patients were diagnosed with myocarditis, and two patients died (one of whom required ECMO support) [27].

In a systematic review of 2020 involving 39 observational studies regarding 622 patients with MIS-C, the most common symptoms were fever and gastrointestinal symptoms. However, skin and mucosal lesions suggestive of KD were also frequently observed. Cardiovascular lesions included decreased cardiac contractility (54.1%), pericardial effusion (22%), and aneurysms (8.1%). Clinial outcomes showed that as many as 71% of patients required hospitalization in the ICU, 60.1% had shock, 4.4% required ECMO support, and 1.7% died. The treatments used included IVIG in first place (76.4%), followed equally by vasoactive drugs (52.3%) and glucocorticosteroids (52.3%). Biologic drugs used included interleukin-1 inhibitor (8.5%) and interleukin-6 inhibitor (6.0%). When differentiating between SARS-CoV-2 and MIS-C virus infection, in addition to fever, which is present in both cases, respiratory symptoms are more frequently observed during SARS virus infection, and in MIS-C, gastrointestinal symptoms are dominant in clinical presentation [5].

A summary of original studies focusing on cardiovascular involvement in MIS-C is presented in Table 1. 

### 2.5. Risk Factors of a Severe Course of MIS-C

MIS-C is a disease with a clinical picture reminiscent of toxic shock, Kawasaki disease, or macrophage activation syndrome. Since the emergence of this new entity, however, researchers have been asking themselves about its underlying cause, which results in much more frequent cardiovascular involvement impairing left ventricular function and creates the need for the hospitalization of PICU patients. The cause of this condition appears to be a cytokine storm that is more severe than in other disease entities in genetically predisposed children, causing vasculitis [28]. At first, there seemed to be a higher risk of cardiovascular involvement and left ventricular systolic dysfunction in patients who had more severe inflammation, so in these patients, a combination of IVIG and glucocorticosteroids as the first line of treatment seemed to be a good therapeutic option. One study showed that a higher initial concentration of cardiac enzymes; higher markers of inflammation (CRP, ferritin, D-dimer); biochemical parameters such as high lactate, creatinine, low albumin, and sodium level; and lower concentrations of platelets, leukocytes, and lymphocytes are statistically significant factors for resistance to the administration of IVIG and the need for steroid treatment [29]. Another study describing risk factors for a more severe course of MIS-C conducted on a larger group of 166 patients showed that a more severe course was seen in older children, with hepatomegaly and splenomegaly, as well as higher CRP, troponin, creatinine, and D-dimers [30]. McArdle et al. performed an international observational cohort study involving as many as 614 patients and performed models in which they evaluated the efficacy of treatment with IVIG alone vs. IVIG and steroids vs. steroids alone. The endpoints referred to the need for inotropic treatment, mechanical ventilation, death, and the dynamics of blood markers of inflammation and organ damage. Their results showed that no one therapy is superior to another, but the authors emphasize the need for further randomized trials on the topic [31]. Another large, retrospective, 2020 US study evaluating risk factors for severe MIS-C involving 1080 patients also found that older age in children (older than 6 years); elevated serum levels of C-reactive protein, troponin, ferritin, D-dimer, brain natriuretic peptide (BNP), N-terminal pro B-type BNP, or interleukin-6; or reduced platelet or lymphocyte counts have a significant impact on disease severity. These risk factors have been linked to both impaired left ventricular systolic function and risk of ICU admission [32]. In patients with MIS-C, the levels of WBC, CRP, D-dimer, and ferritin are worth monitoring, as they tend to be the markers that most promptly reveal the onset of severe MIS-C [9]. 

Other severe complications regard the nervous system. The available literature reports that neurological complications affect up to 34% of patients with MIS-C. Series of case reports from India show that patients can have neurological symptoms including irritability, drowsiness, encephalopathy, stroke, and even coma. MRI is normal in most MIS-C cases. The authors once again emphasize that nervous system changes correlate with the severity of inflammation [33,34,35]. 

### 2.6. Long-Term Complications and Future Directions

There are a few publications in the literature on the long-term effects of undergoing MIS-C, but these are preliminary results due to the rather short follow-up period. At one-year follow-up, laboratory parameters for both inflammation and markers of myocardial damage were found to normalize within an average of 1–4 weeks, but even in 25% of patients, parameters such as D-dimer may remain elevated even one year after the disease. Cardiovascular changes should also retreat within 4 weeks, but up to 18% of patients show mild myocardial dysfunction, and strain parameters normalize within up to 4 months. Changes in the coronary arteries retreat within 8 weeks, but in individual patients, the lesions or aneurysms persist even after a period of 6 months. Other problems include persistent emotional stress, fear of relapse, fear of vaccination, or impaired exercise tolerance in children following MIS-C [36]. 

In an Italian multicenter study from 2022 focusing on long-term cardiovascular outcomes in children with MIS-C with one-month and one-year follow-up periods, it was demonstrated that pericarditis resolved within 6 months and coronaritis within 1 year, and LV dysfunction retreated the fastest, as left ventricular function was already restored to full capacity by the time the child was discharged. However, follow-up also included a cardiac MRI at 9.4 +/− 4.6 months from the onset of the disease, which showed areas of late enhancement, which indicated scarring of myocardial tissue [37]. On the other hand, a 2023 paper evaluating the correlation between changes in echocardiography and electrocardiography and changes in magnetic resonance imaging showed that in the acute phase of the disease, it is possible to show changes in this examination that mainly concern the assessment of left ventricular function and the presence of pericardial fluid, but that these changes regress over time [38].

In a recent article published in May 2024 on the role of MRI in MIS-C, MRI was performed in the study group during the acute phase, showing mainly pericardial fluid, valvular regurgitation, or myocardial edema. These changes retreated in all patients at follow-up, demonstrating the usefulness of MRI as a cardiovascular follow-up study in MIS-C. The literature also reports that MRI is a good method for controlling cardiac lesions in patients treated with anakinra. This test should be performed even in patients in whom no echocardiographic changes were found in the acute phase of the disease [20,39]. Changes in electrocardiograms also dissolved within 6 months of initial diagnosis [40].

Patients should be monitored periodically for up to 6 months when heart MRI should be performed in addition to laboratory tests and echocardiography to evaluate possible myocardial fibrosis. Further follow-up should be determined individually depending on residual cardiovascular changes, but it seems that in children without major pathologies, cardiac follow-up should be performed once a year as in KD [41]. 

Despite the fairly rapid normalization of acute lesions in MIS-C, Farooqi et al. found persistent immune changes and persistent lymphocytosis with increased double-negative T cells, indicating a disruption of the immune system even months after MIS-C [42]. Gelzo et al. examined serum cytokines and 386 genes associated with autoimmune diseases. They found elevated levels of IFNγ and interleukins in MIS-C patients, but with high individual variability. It seems that the determination of cytokines at the time of patient admission would help apply more targeted therapy. This study also showed a decrease in lymphocytes and T, B, and NK cells. Genetic studies showed potentially pathogenic variants in 34 genes related to immune and inflammatory disorders, and more than 80% of patients had one or more variant. These patients should remain under further follow-up, as they may develop autoinflammatory disease in the future [43].

Current observations mainly emphasize decreased exercise tolerance, which indicates the need for exercise testing optimally with ergospirometry for the cardiopulmonary evaluation of these patients, especially if they required hospitalization in the PICU and mechanical ventilation. It is also worth keeping in mind the portion of patients who have persistent coronary dilatation or mild left ventricular dysfunction. The evaluation of lipid disorders, which may, in the future, along with the persistence of subclinical inflammation in some patients, lead to premature atherosclerosis and further complications, also seems to be a good line of research. Some studies also raise the issue of persistent immune disorders, which may lead to the development of autoinflammatory diseases in the future. The psychological burden of this disease should not be forgotten either. It is worth noting the promotion of a healthy lifestyle and the need for physiotherapy in children after MIS-C, especially if they required hospitalization in the PICU to prevent permanent deconditioning and adult complications such as atherosclerosis. It is worth establishing a universal outpatient follow-up protocol to effectively monitor these patients [44].

**Table 1:** Summary of original papers on cardiovascular changes in MIS-C patients.

Authors, Date of Publication	Study Group	PICU	LV Dysfunction	Changes in CA	Pericardial Effusion	Follow-Up
Mileva et al., 2023 [22]	51 patients (73% boys)	9.8%	11%—myocarditis	7.8%	71%	Full recovery at 6-month follow-up
2. Stasiak et al., 2022 [23]	51 patients (51% girls)	31%	27.5%	22.8%	82.35%	6-week follow-up: Pericardial fluid—47% CA changes—19.6 Lower EF—3.9%
3. Uka et al., 2023 [24]	204 patients (69.6% male)	51.5%	36.3%	22.4%	24.9%	Changes in CA—6.6% Pericardial effusion—1.7%
4. Ludwikowska et al., 2023 [25]	498 patients, (63% male)	N/A	25.8%	5.7%	7.7%	5-day follow-up: Changes in CA—5.7% Pericardial effusion—4.5% LV dysfunction—13.9%
5. Cantarutti et al., 2022 [37]	67 patients (60% male)	N/A	65%	35%	66%	Full recovery at 1-year follow-up
6. Dufort et al., 2020 [27]	95 patients (54% male)	80%	52%	7% (CAA)	32%	N/A
7. Avrusin et al., 2023 [30]	166 patients, (59.6% male)	50.6%	30.6%	15.8%	28.8%	N/A
8. Chakraborty et al., 2022 [40]	80 patients (51.2% female)	N/A	33.8%	17.5%	22.5%	At 1-year follow-up CAA—1.3%
9. Farooqi et al., 2021 [42]	45 patients (53.3% male)	N/A	48.9%	15.9%	44.4%	At 4–9-month follow-up LV dysfunction—4.2%

N/A—not applicable, PICU—pediatric intensive care unit, LV—left ventricle, CA—coronary artery, CAA—coronary artery aneurysm, EF—ejection fraction.

## 3. Case Report

**Figure 1:**
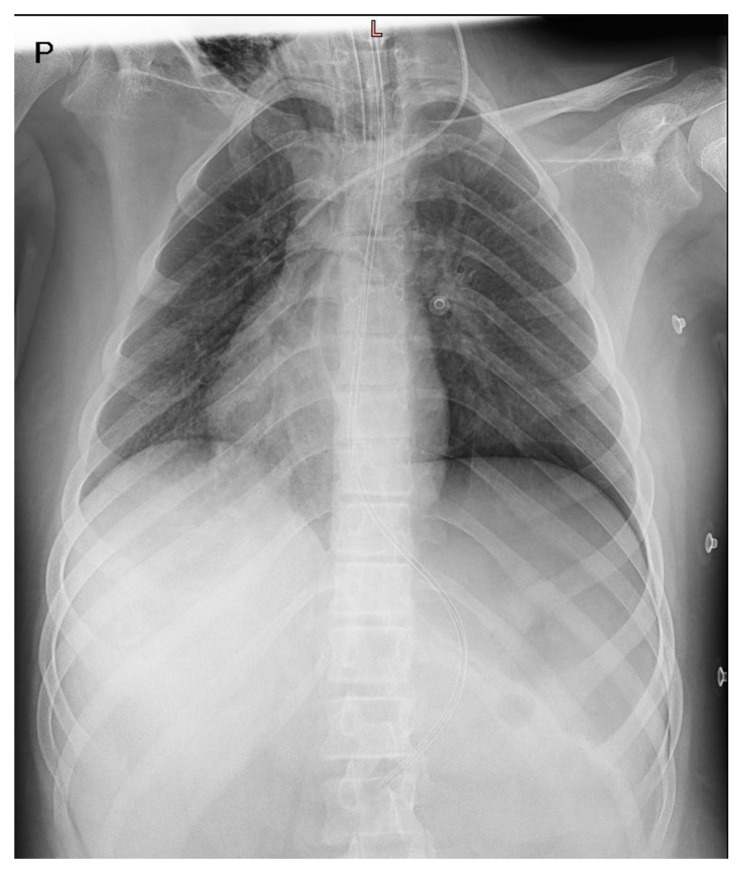
Massive atelectatic and interstitial edematous changes, along with a left pneumothorax and features of mediastinal emphysema in chest X-ray.

**Figure 2:**
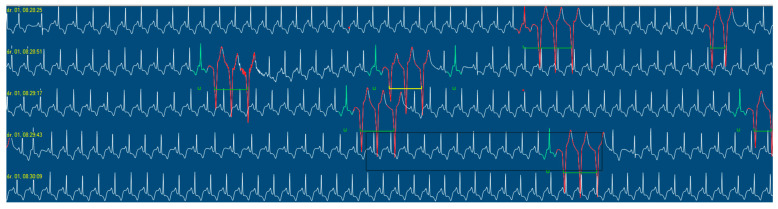
Extract from a 24 h ECG recording revealing complex cardiac arrhythmia in the form of ventricular salvos (in red) with a frequency of 110/min, as well as repolarization abnormalities.

**Figure 3:**
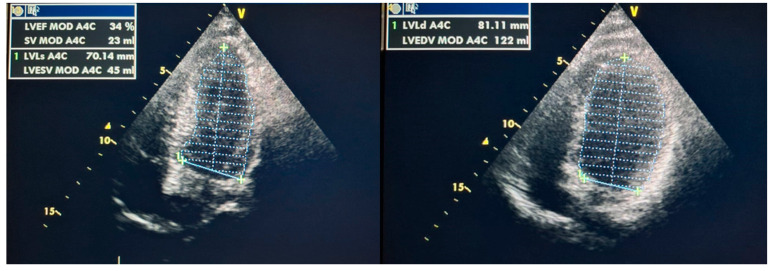
Severe dysfunction of left ventricular function in echocardiography with EF = 34% calculated using the Simpson method.

**Figure 4:**
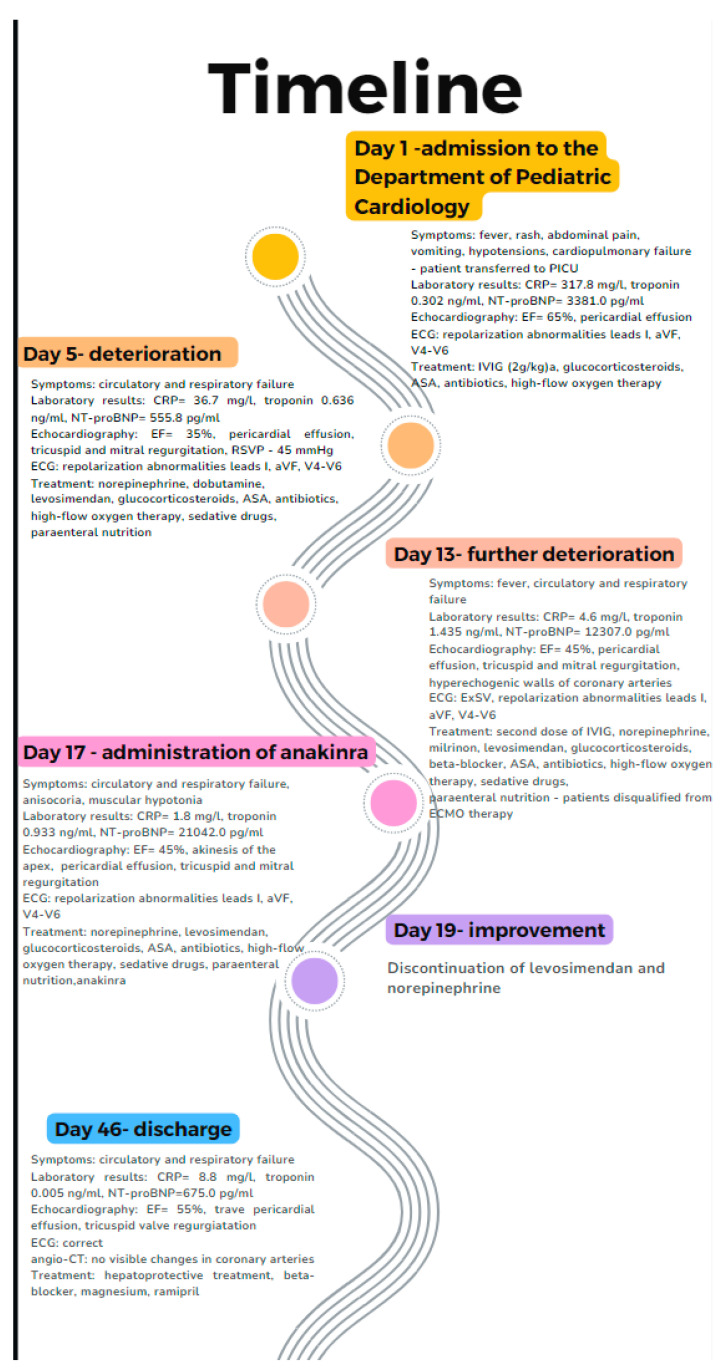
Timeline of patient’s hospitalization including symptoms, laboratory and echocardiographic parameters, as well as treatment.

This case report concerns a 12-year-old male admitted to the Department of Pediatric Cardiology and Rheumatology from the District Hospital due to suspected MIS-C associated with SARS-CoV-2 infection. According to his medical history, the boy presented with fever for 3 days, experienced emesis and sore throat, and reported headache and vertigo. Despite the incorporation of intravenous antibiotic therapy, his clinical condition did not improve; increasing markers of inflammation (high C-reactive protein (CRP), coagulopathy, thrombocytopenia, and escalating markers of heart failure (N-terminal prohormone of brain natriuretic peptide–NT-proBNP)) were observed. Five weeks prior to the onset of symptoms, the male had contact with a person who was infected with the SARS-CoV-2 virus. The result of a PCR test detecting ongoing COVID-19 infection was negative. After admission, his condition rapidly deteriorated, progressive cardiopulmonary failure occurred within hours, and he required a transfer to the pediatric intensive care unit (PICU). In the PICU, the patient’s condition was assessed as extremely severe, with visible respiratory effort, significant hypotension (NIBP 70/40 mmHg), and tachycardia. The boy was intubated and required high-flow oxygen therapy. Antimicrobial therapy was modified, and parenteral nutrition and intensive fluid therapy were incorporated. In regard to circulatory failure, norepinephrine infusion was included. The intravenous infusion of human immunoglobulins (IVIG) (a total dose of 100 g) and systemic steroid therapy were also implemented, as well as acetylsalicylic acid in accordance with MIS-C treatment guidelines. Chest X-ray revealed massive atelectatic and interstitial edematous changes, along with a left pneumothorax and features of mediastinal emphysema (Figure 1). Further deterioration of the patient’s condition was observed in consecutive days with critical arterial blood gas exchange parameters, increasing tachycardia, and oliguria. Follow-up laboratory tests revealed decreasing markers of inflammation; however, markers of myocardial damage had increasing tendencies with NT-proBNP levels of 33,812 pg/mL (Table 2). Ventilatory parameters were modified to extremely high, catecholamine infusions were adjusted as levosimendan, and magnesium sulfate infusions were additionally included alongside norepinephrine and dobutamine infusions. From the 12th day of hospitalization, repeated deterioration was observed, cardiac enzymes increased significantly, and electrocardiographic changes in the form of widened QRS complexes, single premature ventricular and supraventricular beats, complex cardiac arrhythmias in the form of ventricular salvos with a frequency of 110/min, as well as repolarization abnormalities (negative T-wave in V4–V6 leads) appeared in the 12-lead resting ECG as well as in 24 h Holter ECG recordings (Figure 2). Echocardiography revealed progressive deterioration with significant myocardial hypokinesis (ejection fraction—EF of 34%) (Figure 3), significant atrioventricular valve regurgitations, and features of elevated pulmonary pressure (right ventricular systolic pressure—RSVP 45 mmHg); therefore, the patient was consulted with a cardiac surgeon; however, he was disqualified from receiving ECMO therapy. From the 14th day of hospitalization, the onset of anisocoria, a decrease in muscle strength was also observed—electroencephalographic examination showed no seizure activity and brain magnetic resonance imaging (MRI) revealed a subarachnoid cyst (irrelevant finding). An additional intravenous infusion of human immunoglobulins was administered. On the 16th day of treatment, the suspicion of post-inflammatory cardiomyopathy was raised and a beta-blocker, as well as cardioprotective treatment in the form of angiotensin-converting enzyme inhibitor, were included in the therapy. Due to the deteriorating condition of the patient, features of multiple organ failure, as well as persistence of fever, it was decided to incorporate a biologic drug—interleukin-1 inhibitor (anakinra). After the implementation of biological treatment, a gradual improvement in myocardial contractility was observed on echocardiography, and catecholamines were discontinued on day 19. Due to features of hepatic cell damage in laboratory tests, hepatoprotective treatment was started. The patient also required intensive rehabilitation as muscle atrophy and decreased muscle tone were detected in the physical examination. Echocardiographic examination on the day of the patient’s discharge showed preserved global systolic function with EF = 60%, with better contractility of the basal region of the heart in relation to the apical region. Due to the inability to accurately assess the coronary arteries in echocardiography, a coronary angio-CT study was performed, which revealed no abnormalities. Six months after discharge, he also underwent cardiac MRI, which showed no features of myocardial fibrosis and confirmed good myocardial contractility and correct dimensions of the heart cavities. During the two-year follow-up period, the patient has only reported decreased exercise tolerance, with no other cardiovascular symptoms. Resting ECG recordings showed a borderline QTc interval (0.44 s) and prolonged QTc interval to a maximum of 0.475 s in 24 h ECG recording. An exercise test demonstrated good exercise capacity; exercise did not generate arrhythmias or repolarization disorders. The patient remains under further cardiac care. Currently, there is no evidence of impairment in the neurological examination. The uniqueness of the presented case is that the patient developed several serious complications over a short period of time, including persistent and severe myocarditis unresponsive to standard treatment for MIS-C, as well as rare neurological complications. These changes improved spectacularly after treatment with an interleukin-1 inhibitor, highlighting the role of biologic therapy in severe cases of MIS-C. The timeline of the patient’s hospitalization is presented in Figure 4. 

## 4. Material and Methods

The literature review was performed by reviewing the published literature in the MEDLINE, Scopus, and PubMed databases using the words MIS-C, PIMS-TS, children, and SARS-CoV-2 from January 2020 to May 2024 with a special focus on cardiovascular involvement. Inclusion criteria were published peer-reviewed, original research regarding cardiovascular complications in children with MIS-C. We excluded case reports, small (<5 patients) study groups, letters to editors, and studies that focused on a system other than cardiovascular in children with MIS-C.

## 5. Conclusions

The presented case illustrates how diverse the complications and severities of the course of MIS-C are. Cardiovascular changes can include both myocardial and vascular complications, but most of them resolve after the acute phase of the disease. Follow-up of these patients is necessary, as the distant effects of MIS-C in children are unknown. The subclinical scarring of myocardial tissue may lead to cardiac dysfunction and conduction abnormalities in the future. Escalating therapy and introducing biologic drugs into the guidelines allows patients to avoid invasive cardiorespiratory support. Along with the follow-up time, it is important to highlight the changes in the myocardium (especially its post-inflammatory fibrosis), subsequent arrhythmic complications, and those involving the coronary vessels (both changes in the vessels themselves and the possibility of the premature development of atherosclerotic lesions). As shown by individual studies, abnormal laboratory and immunological tests persist in some patients, which carries a risk of atherosclerosis development, thromboembolic changes, as well as the development of autoinflammatory and autoimmune diseases in these patients.

## 6. Future Direction

The clinical follow-up of patients after MIS-C should take place, as with KD, once a year or more often if their clinical condition requires it. The periodic monitoring of ECG recordings, echocardiography, and execution of cardiac MRI seem reasonable. There should be long-term follow-ups of these patients to obtain a complete picture of the course of the disease. Despite the fact that with vaccination and milder courses of SARS-CoV-2 infections, the number of children with MIS-C and the severity of the course of the disease have decreased, pediatricians should remain vigilant, as we are still observing periodic outbreaks of the disease with unexpected courses; it is worth following new recommendations on the diagnosis and therapy of MIS-C.

## Figures and Tables

**Table 2:** Results of laboratory tests in the consecutive days of hospitalization.

Parameter	Day of Hospitalization						Norm
	Day 1 (1st Dose of IVIG)	Day 6	Day 13 (2nd Dose of IVIG)	Day 17 (1st Dose of Anakinra)	Day 31 (Last Dose of Anakinra)	Day 46 (Discharge)	
ALT (U/L)	22	253	107	176	262	48	<41
AST (U/L)	22	88	82	118	72	22	<40
CRP (mg/L)	317.8	36.7	4.6	1.8	1.5	8.8	<5
CK-MB mass (ng/mL)	1.8	1.6	16.4	22.9	3.5	3.3	<5.2
D-dimer (ng/mL)	6057.56	1993.74	681.763	521.72	1152.59	275.22	<500
Ferritin (µg/L)	333.01	569.81	1313.19	1345.42	390.56	334.85	14–124
Fibrinogen (mg/dL)	473.46	225.63	182.98	115.47	278.23	379.85	200–400
Creatinine (mg/dL)	0.86	1.83	0.94	1.07	0,49	0,5	0.58–0.81
Lactate (mmol/L)	6.76	1.63	1.6	1.73	1.86	1.53	0.5–1.6
WBC (thou/µL)	18.65	14.9	16.9	15.36	4.73	10.28	4.5–13
HGB (g/dL)	12.8	13.0	12.6	11.1	11.1	12.0	12.1–16.6
HTC (%)	37.8	40.9	36.7	31.8	32.3	35.5	35–49
PLT (thou/µL)	58	361	387	333	109	366	150–400
LIMPH thou/µL)	0.87	0.69	1.15	0.54	2.88	2.14	1.5–4
NT-proBNP (pg/mL)	33,812.0	555.8	12,307.0	21,042.0	1986.0	675.0	<125
Troponin I (ng/mL)	0.302	0.636	1.435	0.933	0.035	0.005	<0.028

ALT—alanine aminotransferase, AST—aspartate aminotransferase, CRP—C-reactive protein, CK-MB mass—creatine kinase MB mass, WBC—white blood cells, HGB—hemoglobin, HTC—hematocrit, PLT—platelets, LIMPH—lymphocytes, NT-proBNP—N-terminal prohormone of brain natriuretic peptide.

## References

[1] WHO Scientific Statement. Multisystem Inflammatory Syndrome in Children and Adolescents with COVID. https://www.who.int/publications/i/item/multisystem-inflammatory-syndrome-in-children-and-adolescents-with-covid-19.

[2] La Torre F., Taddio A., Conti C., Cattalini M. (2023). Multi-Inflammatory Syndrome in Children (MIS-C) in 2023: Is It Time to Forget about It?. Children.

[3] Multisystem Inflammatory Syndrome in Children (MIS-C) Associated with SARS-CoV-2 Infection 2023 Case Definition|CDC. (n.d.). https://ndc.services.cdc.gov/case-definitions/multisystem-inflammatory-syndrome-in-children-mis-c-2023/a.

[4] Son M.B.F., Son M.B.F., Burns J.C., Burns J.C., Newburger J.W., Newburger J.W. (2023). A New Definition for Multisystem Inflammatory Syndrome in Children. Pediatrics.

[5] Ahmed M., Advani S., Moreira A., Zoretic S., Martinez J., Chorath K., Acosta S., Naqvi R., Burmeister-Morton F., Burmeister F. (2020). Multisystem inflammatory syndrome in children: A systematic review. EClinicalMedicine.

[6] Eleftheriou I., Maritsi D., Lampidi S., Charisi K., Vantsi P., Skourti K., Filippatos F., Amplianitis I., Dimou D., Papadopoulou-Legbelou K. (2022). Decreasing Incidence of the Multisystem Inflammatory Syndrome in Children Over 3 Pandemic Waves. Pediatr. Infect. Dis. J..

[7] McCrindle B.W., Harahsheh A.S., Handoko R., Raghuveer G., Portman M.A., Khoury M., Newburger J.W., Lee S., Jain S.S., Khare M. (2023). SARS-COV-2 variants and multisystem inflammatory syndrome in children. N. Engl. J. Med..

[8] Filippatos F., Tatsi E.-B., Michos A. (2023). Immunology of Multisystem Inflammatory Syndrome after COVID-19 in Children: A Review of the Current Evidence. Int. J. Mol. Sci..

[9] Zhao Y., Yin L., Patel J., Tang L., Huang Y. (2021). The inflammatory markers of multisystem inflammatory syndrome in children (MIS-C) and adolescents associated with COVID-19: A meta-analysis. J. Med. Virol..

[10] Gowin E., Dworacki G., Siewert B., Wysocki J., Lewandowska-Januszkiewicz D. (2022). Immune profile of children diagnosed with multisystem inflammatory syndrome associated with SARS-CoV-2 infection (MIS-C). Cent. Eur. J. Immunol..

[11] Constantin T., Pék T., Horváth Z., Garan D., Szabó A.J. (2023). Multisystem inflammatory syndrome in children (MIS-C): Implications for long COVID. Inflammopharmacology.

[12] Santos-Rebouças C.B., Piergiorge R.M., Ferreira C.d.S., Zeitel R.d.S., Gerber A.L., Rodrigues M.C.F., Guimarães A.P.d.C., Silva R.M., Fonseca A.R., Souza R.C. (2022). Host genetic susceptibility underlying SARS-CoV-2-associated Multisystem Inflammatory Syndrome in Brazilian Children. Mol. Med..

[13] Lin J., Harahsheh A.S., Raghuveer G., Jain S., Choueiter N.F., Garrido-Garcia L.M., Dahdah N., Portman M.A., Misra N., Khoury M. (2023). Emerging Insights Into the Pathophysiology of Multisystem Inflammatory Syndrome Associated with COVID-19 in Children. Can. J. Cardiol..

[14] Basaran O., Batu E.D., Akca U.K., Atalay E., Cuceoglu M.K., Sener S., Balık Z., Karabulut E., Kesici S., Karagoz T. (2023). The Effect of Biologics in the Treatment of Multisystem Inflammatory Syndrome in Children (Mis-C): A Single-Center Propensity-Score-Matched Study. Children.

[15] Henderson L.A., Canna S.W., Friedman K.G., Gorelik M., Lapidus S.K., Bassiri H., Behrens E.M., Kernan K.F., Schulert G.S., Seo P. (2022). American College of Rheumatology Clinical Guidance for Multisystem Inflammatory Syndrome in Children Associated with SARS–CoV-2 and Hyperinflammation in Pediatric COVID-19: Version 3. Arthritis Rheumatol..

[16] Rosario D.C., Ambati S. (2023). Extracorporeal Membrane Oxygenation in Children. StatPearls.

[17] Miller N., Sandhu H.S., Anton-Martin P. (2024). Extracorporeal membrane oxygenation outcomes in multisystem inflammatory syndrome of childhood—An extracorporeal life support organization registry study. Perfusion.

[18] Schwartz S.P., Walker T.C., Kihlstrom M., Isani M., Smith M.M., Smith R.L., McLean S.E., Clement K.C., Phillips M.R. (2020). Extracorporeal Membrane Oxygenation for COVID-19-Associated Multisystem Inflammatory Syndrome in a 5-year-old. Am. Surg..

[19] Maaloul I., Gargouri R., Hadrich Z., Abid L., Kamoun T. (2023). Cardiac Involvement in Multisystem Inflammatory Syndrome in Children. Indian J. Pediatr..

[20] Wu E.Y., Campbell M.J. (2021). Cardiac Manifestations of Multisystem Inflammatory Syndrome in Children (MIS-C) Following COVID-19. Curr. Cardiol. Rep..

[21] Alsaied T., Tremoulet A.H., Burns J.C., Saidi A., Dionne A., Lang S.M., Newburger J.W., de Ferranti S., Friedman K.G. (2021). Review of cardiac involvement in multisystem inflammatory syndrome in children. Circulation.

[22] Mileva N., Vasilev G.H., Ganev B., Chervenkov L., Batselova H., Tzotcheva I., Tomov L., Velikova T., Lazova S. (2023). Cardiovascular Manifestations of Multisystem Inflammatory Syndrome in Children: A Single-Center Bulgarian Study. Medicina.

[23] Stasiak A., Kędziora P., Kierzkowska B., Niewiadomska-Jarosik K., Perdas E., Smolewska E. (2022). Changes in the cardiovascular system in children with pediatric multisystem inflammatory syndrome temporally associated with COVID-19-A single center experience. Int. J. Cardiol..

[24] Uka A., Bressieux-Degueldre S., Buettcher M., Kottanattu L., Plebani M., Niederer-Loher A., Schöbi N., Hofer M., Tomasini J., Trück J. (2023). Cardiac involvement in children with paediatric multisystem inflammatory syndrome temporally associated with SARS-CoV-2 (PIMS-TS): Data from a prospective nationwide surveillance study. Swiss Med. Wkly..

[25] Ludwikowska K.M., Moksud N., Tracewski P., Sokolski M., Szenborn L. (2023). Cardiac Involvement in Patients with Multisystem Inflammatory Syndrome in Children (MIS-C) in Poland. Biomedicines.

[26] Ludwikowska K.M., Okarska-Napierała M., Dudek N., Tracewski P., Kusa J., Piwoński K.P., Afelt A., Cysewski D., Biela M., Werner B. (2021). Distinct characteristics of multisystem inflammatory syndrome in children in Poland. Sci. Rep..

[27] Dufort E.M., Koumans E.H., Chow E., Rosenthal E.M., Muse A., Rowlands J., Barranco M.A., Maxted A.M., Rosenberg E.S., Easton D. (2020). Multisystem Inflammatory Syndrome in Children in New York State. N. Engl. J. Med..

[28] Nakra N.A., Blumberg D.A., Herrera-Guerra A., Lakshminrusimha S. (2020). Multi-System Inflammatory Syndrome in Children (MIS-C) Following SARS-CoV-2 Infection: Review of Clinical Presentation, Hypothetical Pathogenesis, and Proposed Man-agement. Children.

[29] Stasiak A., Perdas E., Smolewska E. (2022). Risk factors of a severe course of pediatric multi-system inflammatory syndrome temporally associated with COVID-19. Eur. J. Pediatr..

[30] Avrusin I.S., Abramova N.N., Belozerov K.E., Kondratiev G.V., Bregel L.V., Efremova O.S., Vilnits A.A., Konstantinova J.E., Isupova E.A., Kornishina T.L. (2023). Determination of Risk Factors for Severe Life-Threatening Course of Multisystem Inflammatory Syndrome Associated with COVID-19 in Children. Children.

[31] McArdle A.J., Vito O., Patel H., Seaby E.G., Shah P., Wilson C., Broderick C., Nijman R., Tremoulet A.H., Munblit D. (2021). Treatment of Multisystem Inflammatory Syndrome in Children. N. Engl. J. Med..

[32] Abrams J.Y., Oster M.E., Godfred-Cato S.E., Bryant B., Datta S.D., Campbell A.P., Leung J.W., Tsang C.A., Pierce T.J., Kennedy J.L. (2021). Factors linked to severe outcomes in multisystem inflammatory syndrome in children (MIS-C) in the USA: A retrospective surveillance study. Lancet Child Adolesc. Health.

[33] Chintha L., Magar S., Vaidya V., Bhartiya S., Mehta K. (2023). Neurological manifestations of multisystem inflammatory syndrome in children associated with COVID-19 in a tertiary care centre. Int. J. Contemp. Pediatr..

[34] Bova S.M., Serafini L., Capetti P., Dallapiccola A.R., Doneda C., Gadda A., Lonoce L., Vittorini A., Mannarino S., Veggiotti P. (2022). Neurological Involvement in Multisystem Inflammatory Syndrome in Children: Clinical, Electroencephalographic and Magnetic Resonance Imaging Peculiarities and Therapeutic Implications. An Italian Single-Center Experience. Front. Pediatr..

[35] Abbati G., Attaianese F., Rosati A., Indolfi G., Trapani S. (2022). Neurological Involvement in Children with COVID-19 and MIS-C: A Retrospective Study Conducted for More than Two Years in a Pediatric Hospital. Children.

[36] Fremed M.A., Farooqi K.M. (2022). Longitudinal Outcomes and Monitoring of Patients with Multisystem Inflammatory Syndrome in Children. Front. Pediatr..

[37] Cantarutti N., Battista V., Stagnaro N., Labate M.E., Cicenia M., Campisi M., Vitali V., Secinaro A., Campana A., Trocchio G. (2022). Long-Term Cardiovascular Outcome in Children with MIS-C Linked to SARS-CoV-2 Infection—An Italian Multicenter Experience. Biology.

[38] Karagözlü S., Ramoğlu M.G., Bayram Ö., Bakhtiyarzada J., Aydın A., Yılmaz M.M., Murt B., Özkan E., İnceli H.B., Gurbanov A. (2023). Cardiovascular manifestations and cardiac magnetic resonance follow-up of multisystem inflammatory syndrome in children (MIS-C). Cardiol. Young.

[39] Maggio M.C., Lembo A., Finazzo F., Alaimo A., Benfratello G.F., Corsello G. (2024). Cardiovascular involvement in children with COVID-19 temporally related multisystem inflammatory syndrome (MIS-C): Can cardiac magnetic resonance arrive to the heart of the problem?. Ital. J. Pediatr..

[40] Chakraborty A., Johnson J.N., Spagnoli J., Amin N., Mccoy M., Swaminathan N., Yohannan T., Philip R. (2022). Long-Term Cardiovascular Outcomes of Multisystem Inflammatory Syndrome in Children Associated with COVID-19 Using an Institution Based Algorithm. Pediatr. Cardiol..

[41] Kumar P., Rajvanshi N. (2023). Multisystem Inflammatory Syndrome in Children (MIS-C): Does it have a Long-Term Impact?. Indian J. Pediatr..

[42] Farooqi K.M., Chan A., Weller R.J., Mi J., Jiang P., Abrahams E., Ferris A., Krishnan U.S., Pasumarti N., Suh S. (2021). Longitudinal Outcomes for Multisystem Inflammatory Syndrome in Children. Pediatrics.

[43] Gelzo M., Castaldo A., Giannattasio A., Scalia G., Raia M., Esposito M.V., Maglione M., Muzzica S., D’Anna C., Grieco M. (2022). MIS-C: A COVID-19-as sociated condition be-tween hypoimmunity and hyperimmunity. Front. Immunol..

[44] Penner J., Abdel-Mannan O., Grant K., Maillard S., Kucera F., Hassell J., Eyre M., Berger Z., Hacohen Y., Moshal K. (2021). 6-month multidisciplinary follow-up and outcomes of patients with paediatric inflammatory multisystem syndrome (PIMS-TS) at a UK tertiary paediatric hospital: A retrospective cohort study. Lancet Child Adolesc. Health.

